# Elucidating the Role of Biofilm-Forming Microbial Communities in Fermentative Biohydrogen Process: An Overview

**DOI:** 10.3390/microorganisms10101924

**Published:** 2022-09-28

**Authors:** Patrick T. Sekoai, Viren Chunilall, Bruce Sithole, Olivier Habimana, Sizwe Ndlovu, Obinna T. Ezeokoli, Pooja Sharma, Kelvin O. Yoro

**Affiliations:** 1 Biorefinery Industry Development Facility, Council for Scientific and Industrial Research, Durban 4041, South Africa; 2 School of Chemical Engineering, University of KwaZulu-Natal, Durban 4041, South Africa; 3 Department of Biotechnology and Food Engineering, Guangdong Technion-Israel Institute of Technology (GTIIT), Shantou 515063, China; 4 Department of Biotechnology and Food Technology, Faculty of Science, University of Johannesburg, Johannesburg 2092, South Africa; 5 Unit for Environmental Sciences and Management, North-West University, Potchefstroom 2520, South Africa; 6 Environmental Research Institute, National University of Singapore, 1 Create Way, Singapore 138602, Singapore; 7 Energy Technologies, Lawrence Berkeley National Laboratory, Berkeley, CA 94720, USA

**Keywords:** biohydrogen, biofilms, fermentation, biofuels, renewable energy

## Abstract

Amongst the biofuels described in the literature, biohydrogen has gained heightened attention over the past decade due to its remarkable properties. Biohydrogen is a renewable form of H_2_ that can be produced under ambient conditions and at a low cost from biomass residues. Innovative approaches are continuously being applied to overcome the low process yields and pave the way for its scalability. Since the process primarily depends on the biohydrogen-producing bacteria, there is a need to acquire in-depth knowledge about the ecology of the various assemblages participating in the process, establishing effective bioaugmentation methods. This work provides an overview of the biofilm-forming communities during H_2_ production by mixed cultures and the synergistic associations established by certain species during H_2_ production. The strategies that enhance the growth of biofilms within the H_2_ reactors are also discussed. A short section is also included, explaining techniques used for examining and studying these biofilm structures. The work concludes with some suggestions that could lead to breakthroughs in this area of research.

## 1. Introduction

As the world is pushing for the intensification of clean and sustainable technologies in order to reduce the problems caused by fossil fuels (greenhouse gas emissions, environmental issues, escalating energy prices, etc.), scientists are constantly searching for alternative fuels that could serve as suitable replacements. Hydrogen has been proposed as an ideal fuel option due to its outstanding properties—it is considered the cleanest fuel as it produces water and oxygen when combusted [1]. Hydrogen also has a high energy content (122 kJ/g) that is 2.5 times higher than hydrocarbons and it can be converted into electricity in fuel cells [2]. Nevertheless, renewable and scalable H_2_ technologies, such as water electrolysis are energy-intensive, and costly [2]. For this reason, alternative approaches, such as biological-based H_2_ production, are explored to surpass these limitations [3].

The commercialization of the biohydrogen production process is still plagued by low process yields—the empirical yields are around 30–50% of the theoretical yield [4]. Secondly, biomass feedstocks must undergo vigorous pretreatment methods, and these techniques are energy-intensive and costly [5]. Another major challenge is that most biohydrogen production studies are conducted at the bench-scale and the process dynamics for the pilot-scale are not well-understood in the literature [6].

Most biohydrogen enhancement studies have focused on optimizing the operational setpoint conditions for the past decade. Herein, the H_2_-producing parameters, such as pH, temperature, substrate concentration, and hydraulic retention time (HRT), are optimized using various mathematical tools, such as response surface methodology (RSM), artificial neural network (ANN), etc. [7]. Other strategies that have been widely explored in the literature include the use of additives/growth nutrients that target the predominant H_2_-producing monocultures of *Clostridium* species and the pretreatments of biomass that serve as substrates during biohydrogen fermentation [8,9].

Despite these efforts, the scalability of biohydrogen production has not yet been achieved, implying that other innovative and robust bioaugmentation methods must be implemented to achieve this goal. Research is now geared towards understanding the microbial ecology of H_2_-producing microorganisms in mixed communities to fully elucidate the synergistic interactions between the active H_2_-producers (e.g., *Clostridium* sp.) and non-active H_2_-producers (e.g., *Enterobacter* sp., *Bacillus* sp., etc.). These bacterial communities have been shown to co-exist during the fermentative biohydrogen process leading to the formation of biofilms—structures composed of aggregated heterogeneous species encapsulated within layers of extracellular polymeric substances (EPS) that serve as the biofilm “binder” [10]. The presence of biofilms offers numerous benefits to the biohydrogen production process, such as improved biomass digestibility, consumption of O_2_ within the reactor, inhibition of toxins, elongation of the H_2_ fermentation periods, maintenance of optimal pH, and the use of different carbon sources [11].

Moreover, in-depth knowledge about the dominant biofilm-formers during H_2_ fermentation can lead to the development of robust H_2_ biotechnological processes as these microbial species can be used as model organisms in H_2_ enhancement studies. This will also enable scientists to better understand the physiological conditions of key model organisms and help to elucidate the links between their ecosystem and nutritional needs [12]. Our current knowledge of biofilms has mostly been derived from research conducted in public health, food technology, and wastewater treatment [13].

Given the complexity of the biofilm structure, the functions of the microbial entities within the biofilm have not been fully elucidated in biohydrogen production studies, as evidenced by the few published studies [12,14]. Therefore, this work provides an overview of the heterogenous biofilm-forming communities that participates during biohydrogen production processes to demonstrate the significance of microbial diversity during biohydrogen fermentation, as this leads to synergistic interactions amongst the various phylum groups and the role of these microbial species in the enrichment of H_2_ process yields. The microbial biofilm enriching methods, such as the use of: (i) biocarriers, (ii) optimal reactor designs, (iii) micronutrients, and (iv) inoculum, are also discussed in this review paper. The work also explores the biotechnological methods that are used for examining and studying these biofilm-forming structures. Finally, the review provides some suggestions that could help develop engineered biofilms in biohydrogen production studies.

## 2. Shedding Light on Microbial Biofilms

Microbial biofilms, due to their ubiquitous nature and complex structure, have attracted significant attention over the past decades. These multicellular organisms serve as drivers and/or regulators of the “global microbiome” and significantly impact humans, plants, and animals [15]. Biofilms are architectural colonies consisting of diverse microbial communities, and these heterogeneous species firmly attach to surfaces (biotic and abiotic) and are enclosed in a self-produced EPS, which accounts for ~90% of the biomass [16]. The EPS is the main component of biofilms because it contributes to their unique features such as porosity, hydrophobicity, mechanical stability, tolerance to external stresses, and density [17]. Furthermore, it consists of essential macromolecules such as carbohydrates, lipids, polysaccharides, proteins, nucleic acids, and other molecules [18]. Within the biofilm structure, a thriving community enables synergist interactions amongst different bacterial sub-populations leading to cell-to-cell interactions and DNA exchange [18].

**Figure 1:**
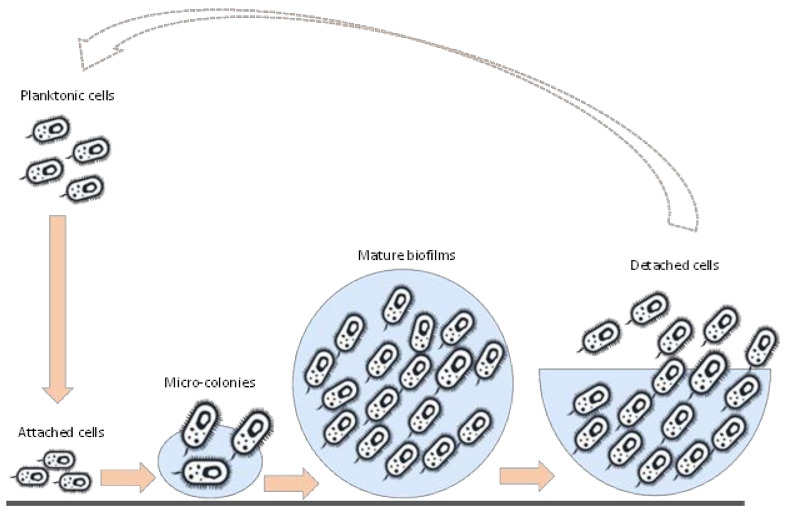
A schematic diagram showing the life cycle of microbial biofilms.

Additionally, the regulation of gene expression is typically impacted by fluctuations in cell-population density and is known as quorum sensing, a feature in which bacterial cells produce and release chemical signal molecules known as autoinducers. The level of released autoinducers increases as a function of cell density in a given environment, allowing for the regulation of key genes and providing bacterial cells with a phenotypic edge [19]. Compared to their planktonic counterparts, sessile cells embedded within biofilm structures are resistant to environmental stresses such as extreme temperatures, pH, nutrient deprivation, ultraviolet radiation, high salinity, antibiotics, and chemicals [20]. Consequently, biofilms exhibit phenotypic and genetic traits distinct from planktonic cells [21]. The formation of sessile biofilms involves a multi-step process that starts with the irreversible attachment of planktonic cells to surfaces, followed by the maturation of the aggregated micro-colonies under optimal growth conditions [22]. The final stage is an established biofilm with diverse microbial communities [23]. This is succeeded by the biofilm detachment process, which can occur at any stage of the biofilm’s lifecycle and may lead to the release of planktonic cells, aggregated cells, and biofilm-produced chemicals [24]. The detachment process can be triggered by the biofilm’s lifecycle or external factors, such as hydrodynamic shear conditions, physical contact, and chemical disinfectants [25,26]. A schematic diagram illustrating the typical lifecycle of biofilms is presented in Figure 1. Since biofilms are also known to regulate the biogeochemical cycling processes in soil and water [26], they have been engineered and applied in various biotechnological processes to remove pollutants in wastewater and solid waste and produce high-value-added products, such as biofuels and biochemicals through biocatalytic processes [25].

## 3. An Overview of the Role of Biofilms in Biohydrogen Fermenter Systems

In recent years, bacterial biofilms have been shown to have remarkable effects on biohydrogen fermenter systems. These versatile and aggregated microbial communities confer several benefits compared to planktonic cells. These include high biomass density, high substrate utilization, reduced HRTs, synergistic interactions amongst various bacteria, tolerance against toxins, maintenance of the optimal pH, and high biohydrogen yields [27,28].

Mei et al. [29] studied the operational conditions that lead to the formation of biofilms in the packed-bed reactor. The HRT of 12 h, substrate concentration of 15 g/L, and an inoculation ratio of 35% favored the biofilm formation. Bacterial groups belonging to *Clostridium* and *Lactobacillus* were the abundant biofilm-forming species, and these results coincide with literature as *Clostridium* sp. are the most dominant H_2_ producers [29]. The occurrence of *Lactobacillus* was also important as it participates in lactic acid production, and this metabolite is later converted to acetic acid by *Clostridium* sp. under anoxic microenvironments [29,30]. Furthermore, the presence of these bacterial species is advantageous as this leads to co-metabolism during the acidogenic fermentation process. More importantly, *Lactobacillus* was effective in prolonging the biohydrogen fermentation as it is more tolerant to acidic conditions than *Clostridium* sp. This synergist interaction boosts the acclimatization of biohydrogen-producing biofilms within the reactor [31].

It was also revealed in another biohydrogen study that the biofilms not only increased the H_2_ content within the reactor but also aided in the degradation of inhibitors [32]. In this work, fermentation inhibitors, such as 5-hydroxymethyl furfural (>40% of the initial quantity detected) and furfural (>70% of the initial amount detected) were successfully degraded by the heterogenous biofilm-forming populations within the H_2_ reactor [32]. The biofilm community structure showed the abundance of *Bacillus* and *Clostridium*, and these species are associated with acidogenesis, which is the main biohydrogen-producing step. Interestingly, the non-biohydrogen species (e.g., *Pseudomonas*) were also beneficial in this fermentation process as these consortia were shown to be effective in the degradation of H_2_ inhibiting compounds, such as aromatic compounds [32]. Likewise, ammonia inhibition is another process issue in biohydrogen fermentation as it proliferates within the reactor and competes with the H_2_-producing pathways. Therefore, biofilms are beneficial in the biohydrogen process as they have been reported to withstand NH_4_^+^ concentrations (<0.14 g/L) during the fermentation process, thus leading to process stability [33].

Zhang et al. [34] studied the biosynthesis of biohydrogen in a continuous stirred tank reactor (CSTR) and anaerobic fluidized bed reactors (AFBRs) using suspended, granular, and biofilm sludge at 37 °C and pH 5.5. The use of sessile microorganisms was beneficial, as more than 10-fold of H_2_ was attained by the granular sludge in the CSTR, and more than 20-fold of H_2_ was achieved by the biofilms in the AFBRs. Using granular sludge and biofilm enhanced biomass retention instead of suspended cells, leading to biomass washout [34]. Acidogenic biofilms were also shown to strengthen substrate utilization as more than 80% of chemical oxygen demand (COD) was converted into H_2_ and its constituents, i.e., volatile fatty acids (VFAs) such as acetic acid, butyric acid, and propionic acid [35]. Nevertheless, maintaining the optimal pH range (using pH sensors and actuators or manual pH control) is essential as acidogenic biofilms are sensitive to VFAs because these metabolites decrease the pH, leading to the growth of H_2_-scavenging methanogens [36].

Odom and Wall [37] conducted a batch process consisting of biofilm communities that are tolerant (*Cellulomonas* strain ATCC 21399) and non-tolerant (*Rhodopseudomonas capsulata*) to oxygen during biohydrogen production. *Cellulomonas* strain ATCC 21399 was strategically applied to convert cellulose into simple monomers such as succinate, acetate, lactate, and formate, and these were later metabolized by *Rhodopseudomonas capsulatus* to generate biohydrogen. During the fermentation process, the biohydrogen increased from 4.6 to 6.2 mol H_2_/mol glucose. A recent study by García-Depraect et al. [14] revealed that lactic acid bacteria (LAB) can establish synergistic interactions with the core H_2_-producers such as *Clostridium* sp. In this work, the LAB was used in conjunction with H_2_-producing consortia to enrich the H_2_ yield at an optimal HRT of 12 h. These results may help scientists identify the set of microbial species that can lead to the optimization of H_2_ process yields. In another syntrophic metabolism of *Clostridium butyricum* and *Rhodobacter sphaeroides*, an H_2_ yield of 0.6 mL H_2_/mol glucose was obtained during biohydrogen fermentation, whereas the use of monocultures of *C. butyricum* led to a minimum H_2_ yield of 0.5 mL H_2_/mol glucose [38]. It was further shown that pH regulation is important in co-culture systems, i.e., the H_2_ yield was decreased upon adjusting the pH to 6.5. When a microbial consortium consisting of starch degrading bacteria (*Rhodobium marinum*, *Vibrio fluvialis*, and *Proteus vulgaris*) and photoautotrophic species (*Dunaliella tertiolecta* ATCC 30929 and *Chlamydomonas reinhardtii* IAM C-238) was used in the biohydrogen production process, a maximum H_2_ yield of 39.1 mmol H_2_/L medium was obtained, suggesting that the mixed microbial strains had a positive synergistic relationship for biohydrogen production [38].

## 4. Biofilm Enrichment Methods Applied in Biohydrogen Fermenter Systems

### 4.1. Carrier Materials for Biofilm Growth

Many different carrier materials have been tested for the enrichment of acidogenic biofilms during biohydrogen fermentation studies (Table 1). Organic carriers widely used in biohydrogen fermentation include activated carbon, expanded clay, and organic gels [39,40]. Silica, ceramic beads, zeolites, acrylamide, polyethylene, and polyvinyl chloride are common inorganic carriers used in biohydrogen production studies [41,42]. The shapes of these carrier materials are cylindrical, granular, or spheroidal, varying from 1.5 to 25.0 mm, while their densities range from 0.5 to 2.0 g/cm^3^ [27]. The carriers are selected based on their hydrophilicity, non-biodegradability, non-toxicity to bacterial species, non-reactivity to chemicals, solid mechanical stability, affordability, high biomass retention, roughness, low surface energy, and good permeability [43]. Such physicochemical properties are crucial for bacterial biofilms’ initial adhesion and maturation within the H_2_ reactor [44]. As a result, inorganic carriers are preferred because of their superior mechanical stability compared to their organic counterparts [45]. It was recently shown that a long-term H_2_ production process (50 days) could be achieved using chlorinated polyethylene (CPE) and zeolite as microbial and nutritional carriers in a hybrid reactor that was operated under semi-continuous conditions [46]. Interestingly, the hybrid-Fe reactor coupled with zeolite could produce H_2_ for up to 72 days without any process instabilities. The acetate pathway (the main H_2_ metabolic route) was induced by the synergistic biofilms [46]. These outstanding results are attributed to the superior properties of CPE and zeolite. CPE provides a suitable roughness surface and high porosity for microbial attachment, resulting in the growth of acidogenic biofilms within the H_2_ reactor, as was corroborated by the SME images [46]. Meanwhile, zeolite is widely used in anaerobic digestion processes as a carrier material because it consists of essential H_2_-enriching micronutrients, such as Ca, Al, Mg, and Na [46]. The presence of Fe also enhances the fermentation process as it boosts the hydrogenases—these are the key enzymes that regulate the H_2_-producing pathways [46].

Other studies that used carrier/support materials in biohydrogen fermentation systems also showed remarkable outcomes, with some reports producing a maximum H_2_ yield that is 4-fold [47] and 25-fold [48] more than the suspended cultures. Herein, the carriers helped the acidogenic biofilms suppress the H_2_-consumers that are concomitantly produced with the H_2_ during acidogenesis [49]. They also prolonged the biohydrogen fermentation periods, resulting in low VFA production [50,51].

Based on these scientific reports, it can therefore be shown that the use of “acidogenic biofilm engineering” technologies could provide many breakthroughs in the area of biohydrogen process development as substrate pretreatment accounts for more than 60% of the overall biohydrogen costs—the biofilms could help in reducing the high costs as some acidogens exhibit cellulolytic activities, and these could be optimized by the biocarriers.

### 4.2. Inoculum with Heterogenous Species for Synergistic Biofilm Interactions

In biohydrogen production studies, mixed sludges are favored due to their non-stringent bioprocess requirements, as H_2_ can be produced under non-sterile conditions at various conditions [66]. Furthermore, acidogenic fermentation involving the sludge is usually preferred for pilot-scale demonstrations as they are easier to operate and control than monocultures [67]. In contrast, pure cultures pose a challenge in biohydrogen fermentation due to their specific requirements for pure sugars (glucose and fructose), thus escalating the biohydrogen production costs. In addition, they must be cultivated under sterile conditions and are prone to contamination [68].

Sludges also consist of biofilms with heterogeneous species, which co-exist to provide various metabolic functions that benefit the biohydrogen process [69]. Bacterial species including *Clostridium*, *Bacillus*, *Enterobacter*, *Prevotella*, *Citrobacter*, *Klebsiella*, *Enterobacter*, *Escherichia coli*, *Lactobacillus*, etc., have been identified in biohydrogen production studies involving anaerobic mixed sludge as the inoculum source [12,70]. The presence of these communities within the H_2_ reactor leads to synergistic associations, enabling bacterial communities to provide different metabolic roles during the fermentation process [71,72]. This phenomenon was observed when the inactive H_2_-producing strains (*Enterobacter* sp.) contributed to H_2_ production alongside the active H_2_ producers (*Clostridium* sp.) [73]. *Enterobacter* sp. was resistant to VFAs and maintained the pH [73]. In a CSTR, the strict anaerobes (*Clostridium*) and facultative anaerobes (*Enterobacter*) established a synergistic relationship to enhance the biosynthesis of H_2_ [74]. While the *Clostridium* predominantly contributed to H_2_ production, *Enterobacter* assisted in consuming O_2_ within the reactor [74].

Similarly, *Bacillus thermoamylovorans* served as a symbiotic partner for biomass conversion when co-cultured with *C. butyricum* [75] and *C. beijerinckii* [76] in anaerobic batch fermenters treating brewery waste. In both studies, *B. thermoamylovorans* reduced the lag phase; contributed toward O_2_ depletion, thus fostering the production of H_2_ as *Clostridium* growth was the main species detected during the optimal H_2_ production stage [75,76].

In other biofilm studies, it was observed that seed sludge also comprises bacterial groups with high hydrolytic capabilities, thus forming a metabolic synergy with H_2_ producers [77]. Isolates such as *Lactobacillus plantarum*, *Olsenella genomo* sp., and *Bifidobacterium* sp. were all characterized during the production of H_2_ in a starch-fed fermenter [78,79]. These three facultative heterofermentative LAB can hydrolyze starch to produce lactate and some traces of acetate but not H_2_ [14,80]. However, these were abundant during hydrolysis of carbohydrate-rich feedstocks and H_2_ production, confirming their amylolytic activity [14,80].

It is noteworthy to highlight that raw sludge must undergo pretreatments as it contains diverse microbial communities, including the H_2_-consuming methanogens. For this reason, the suppression of archaeal communities is crucial for attaining the H_2_ fermentation process [81]. However, this step must be carefully conducted so that bacteria that are beneficial to the H_2_ process are not entirely suppressed due to the harsh pretreatment.

### 4.3. Optimal Reactor Design for Biofilm Growth

As shown in Table 1, different reactor designs are applied in biohydrogen fermentation processes. Studies targeting the enrichment of multispecies biofilms evaluate several factors such as the reactor’s geometry, diameter and height, the reactor type, the substrate treatment capacity of the reactor, and the reactor’s ability to retain biomass and biocarriers during acidogenic fermentation [82]. The up-flow anaerobic sludge blanket reactor (UASBR) has excellent self-immobilization capabilities as bacterial cells forms aggregates without the need for a support/carrier medium leading to high biomass retention and high substrate conversion efficiency [83], this is usually achieved by applying appropriate up-flow velocities, and this reactor can be operated at mesophilic and thermophilic conditions [84]. UASBR is constructed in horizontal and vertical forms and used at short HRTs—this is ideal for acidogenic biofilm communities as they optimally produce H_2_ at short HRTs [85]. The stirring function with the rinsing flow is used without the need for recirculation streams [86]. Likewise, the anaerobic fluidized bed reactor (AFBR) is the most efficient reactor design for biomass retention as it uses various biocarriers to attach to bacterial cells [86]. In AFBR, the bacterial communities undertaking H_2_ production combine to form layers of diverse biofilms with different sizes, geometry, density, and hydrodynamic behavior [87]. Therefore, substrates attach to these biofilms leading to biofilms with high densities and rich nutrients [88]. Continuous stirred tank reactors (CSTRs) are also common in biohydrogen studies due to their high biomass retention abilities and substrate conversion efficiency [89]. Batch systems are widely used mainly due to their simplicity and affordability—they are ideal for preliminary H_2_ investigation studies but not suitable for the cultivation of biofilms during H_2_ fermentation.

### 4.4. Micronutrients for Biofilm Growth

The growth of acidogenic biofilms primarily depends on the carbon source used during acidogenic fermentation. For years, glucose and sucrose have been used as the main carbon source when enriching the acidogenic biofilm-formers, as evidenced by some of the fermentation studies outlined in Table 1. The reliance on these monomeric sugars is not sustainable as feedstocks account for >50% of the overall H_2_ costs [90]. Recent studies focus on biomass residues to circumvent this issue because these carbon materials are readily available, affordable, and considered waste [91]. The main carbon sources should be used in conjunction with the micronutrients (e.g., Ca, Cu, Mg, Ni, Mn, Pb, Zn, etc.) in order to boost the H_2_-regulating enzymes and metabolic pathways [92]. Similarly to organic wastes, wastewater from the brewery and other food processing industries consists of the micronutrients mentioned above and could play a pivotal role in reducing H_2_ production costs [93]. Moreover, using industrial effluents will not only boost the advancement of biohydrogen process technology but also assist in alleviating environmental pollution.

## 5. Biofilm Structural Analysis in Biohydrogen Reactors

The morphological assessment of biofilms is carried out using either spectroscopic- or microscopic-based techniques [94,95]. The advancement in these methods has also enabled the detection of the biofilms’ components, such as lipids, proteins, extracellular DNA, and humic substances [96]. These structural observations can help researchers gain knowledge about (i) the localization and shape of biofilm-forming species (e.g., rod-shaped and/or cocci), (ii) how biomass pretreatment can be improved, and (iii) feedstocks that are easily hydrolyzed and suitable for acidogenic biofilms, and (iv) information about the process performance [22]. Techniques such as scanning electron microscopy (SEM), fluorescence in-situ hybridization (FISH), three-dimensional excitation-emission matrix (3D-EEM) fluorescence spectroscopy, and confocal laser scanning microscopy (CLSM) are widely used for biofilm analysis [83].

## 6. Molecular-Based Analysis of Biofilm Communities in Biohydrogen Reactors

The study of microbial species and their activities within biofilms can also be achieved through molecular techniques, which until recently have become accessible due to technological advances in next-generation sequencing (NGS) technologies and their affordability, yielding large-data sets for bioinformatic investigations [97]. From the extracted genomic DNA (gDNA) of the biomass within biohydrogen reactors, metagenomic sequencing can be performed following the amplification of the phylogenetic marker 16S rRNA gene. Such an approach would allow the investigation of the presence and abundance of specific microbial groups during the biohydrogen process. In the same light, the extraction of RNA from biofilm samples and the subsequent amplification of 16S rRNA genes from complementary DNA (cDNA) could also shed light on the active/dominant microbial populations during biohydrogen production processes. Combining the abovementioned sequencing techniques focused on identifying total and active microbial populations within studied biofilms should provide useful fundamental information on the underlying fitness of targeted microbial species responsible for biohydrogen production in relation to the presence and activity of other microbial groups. Such basic information could provide the basis for optimizing and engineering biofilms’ systems for biohydrogen processes.

Molecular techniques targeting specific genes implicated in biohydrogen production can be achieved through reverse transcription polymerase chain reaction (RT-PCR), which combines reverse transcription of RNA into cDNA followed by the amplification of specific DNA targets using polymerase chain reaction (PCR). Such a technique could help assess and optimize processing conditions and engineer functional biofilm systems for biohydrogen production. This method was successfully employed to elucidate the role of ammonia-oxidizing microorganisms in acidic forest habitats by studying the *amoA* gene [98]. Whole genome sequencing allowing for both the metagenome-assembly of the microbial community and the recovery of metagenomes-assembled genomes (MAGs) could provide interesting genomic insights as well as provide a reference for comparative studies with isolate genomes derived from strains used for inoculating engineered biofilms. When combined with meta transcriptomic analyses, engineered systems’ gene expression profiles could further be explored to optimize experimental conditions for biohydrogen production [99]. When applied in the context of biofilms in biohydrogen processes, such techniques could provide fundamental knowledge of specific functional genes and their corresponding hosts in engineered systems.

## 7. Conclusions and Recommendations

Biofilms are metabolically complex and phylogenetically diverse species that play various metabolic functions during the biohydrogen fermentation process, as demonstrated in this review. These bacterial aggregates consist of active H_2_-producers and non-active H_2_-producers, which provide many beneficial traits such as biohydrogen fermentation, biomass conversion, and inhibition of toxins. The enrichment of acidogenic biofilms is highly dependent on factors such as the carrier type, reactor design, and micronutrients, as shown in this work. Nevertheless, there are many unknowns regarding the co-metabolic pathways of acidogenic biofilm-forming communities. Therefore, the following recommendations are proposed for future studies in this research field.

- An extensive understanding of the key biofilm-forming assemblages during the acidogenic fermentation will help researchers develop microbial characterization strategies (biochemical and molecular tools) that are more effective in identifying these complex and fastidious species. This will be instrumental in developing biofilm starter cultures, consisting of different monoculture biofilms with synergistic/symbiotic abilities, and these can be used as model organisms for biohydrogen optimization studies, with the possibility of scaling up the process.
- The EPS remains the key component of microbial biofilms as it houses diverse phylum communities. It has been quantified in some reports but not to its total capacity, particularly when elucidating its roles in forming acidogenic biofilms. Therefore, it is essential to address these knowledge gaps as this will lead to many scientific breakthroughs in biohydrogen process development.
- Further studies should be conducted to identify the optimal biocarrier materials, biocarrier shapes, and reactors coupled with biocarriers to confer better biofilm growth. Nanoparticles and coagulants have recently been suggested as these materials promote better aggregation and chemical bonds between various biofilms [83].
- Integrating biohydrogen processes with other technologies (e.g., biogas and bio-electrochemical systems), under the concept of “circular economy”, could advance this technology as some of these biotechnological processes have already reached pilot-scale, implying that they have a potential for large-scale. The biohydrogen process could be used as an initial biomass conversion/hydrolysis step followed by using acidogenic metabolites in the biogas or bioelectricity production.

## Figures and Tables

**Table 1:** Types of carrier materials used in biohydrogen fermenter systems to promote biofilms.

Carrier Material	Carrier Size (mm)	Substrate	Inoculum	Reactor Type	Operational Setpoint Conditions			H_2_ Yield	Effects of Biofilms on Process Performance	Reference
					Temp (°C)	pH	Time (d)			
Mixed polymers	5.0	Trace metals	*Rhodopseudomonas faecalis*	CSTR	35	7.0	25	3.24 mol H_2_/mol acetate	70% substrate utilization was achieved.	[52]
Activated carbon	–	Molasses	Mixed cultures	CMISR	35	4.06–4.28	45	130.57 mmol H_2_/mol	The formation of toxins was reduced.	[53]
PEG	3.0	POME	Mixed cultures	UASBR	37	7.0	6.25	0.632 L H_2_/L/h	The process exhibited a high H_2_ yield and process stability.	[54]
Silicone gel	3.0–4.0	Sucrose	Mixed cultures	DTFBR	40	6.0	12.5	1.20 mol H_2_/mol sucrose	There was a superior H_2_-producing performance.	[55]
Pumice stone	1.0–5.0	Sucrose	Mixed cultures	UASBR	55	5.5	1.0	308 mL H_2_/d	There was a 6-fold H_2_ increase.	[56]
Ceramic ring	7.0	Sucrose	Mixed cultures	UASBR	55	5.5	1.0	386 mL H_2_/d	There was a 6-fold H_2_ increase.	[56]
Expanded clay	2.8–3.35	Glucose	Mixed cultures	AFBR	30	6.40	0.33	2.49 mol H_2_/mol glucose	The H_2_-producing pathways were favored.	[57]
Sodium alginate and polyaniline nanoparticles	3.0	Dairy wastewater	Mixed cultures	Batch	35	5.5–6.0	8.3	54.5 mL H_2_/g VS	There was a 285% increase in H_2_ yield.	[58]
Clay and activated carbon	–	Sucrose	Mixed cultures	Batch	39	8.08	16	–	H_2_ could be produced for up to 15 days.	[59]
Coconut coir	–	Nutrient broth	Mixed cultures	Batch	37	7.0	1.0	2.83 mol H_2_/mol hexose	H_2_ was produced for 40 days under non-sterile conditions.	[60]
Sodium citrate	–	Activated sludge	Mixed cultures	Batch	37	7.0	2.0	28.6 mL/g-VS_added_	The H_2_ yield was increased by 346.9% and the lag phase was also shortened.	[61]
Chlorinated polyethylene	–	Trace metals	Mixed cultures	Batch	35	5.5	9.0	27.2 mL H_2_/g glucose	H_2_ could be optimally produced for up to 72 days.	[46]
Zeolite	–	Trace metals	Mixed cultures	Batch	35	5.5	9.0	32.3 mL H_2_/g glucose	H_2_ could be optimally produced for up to 72 days.	[46]
Sodium alginate, chitosan, and SiO_2_	–	Food waste	Mixed cultures	CSTR	37	5.0–6.0	35	1.75 mol H_2_/mol substrate	A 99.4% substrate utilization efficiency was accomplished.	[62]
Granular activated carbon	2.0–3.0	POME	Mixed cultures	AFBR	60	6.0	7.0	1.24 mol H_2_/mol sugar	The H_2_-producers coexisted with the non-H_2_ species.	[63]
Polyvinyl alcohol	–	Trace metals	*Rhodopseudomonas palustris*	Photoreactor	28	7.0	20	15.74 mL H_2_/g/h	A 43% substrate conversion efficiency was achieved.	[64]
Alginate and TiO_2_	2.0–5.0	Glucose	*Escherichia coli*	Batch	37	7.0	3.0	2.8 mmol H_2_/mmol glucose	The presence of oxygen could not inhibit the process.	[65]

–: Not available; AFBR: anaerobic fluidized bed reactor; continuous stirred tank reactor; CMISR: continuous mixed immobilized sludge reactor; CSTR: continuous stirred tank reactor; DTFBR: draft tube fluidized bed bioreactor; PEG: polyethylene glycol; POME: palm oil mill effluent; UASBR: up-flow anaerobic sludge blanket reactor.

## Data Availability

Not applicable.

## Abbreviations

3D-EEM, three-dimensional excitation-emission matrix; 16S rRNA, 16S ribosomal ribonucleic acid; AFBR, anaerobic fluidized bed reactor; ANN, artificial neural network; cDNA, complementary DNA; CLSM, confocal laser scanning microscopy; CPE, chlorinated polyethylene; COD, chemical oxygen demand; CSTR, continuous stirred tank reactor; DNA, deoxyribonucleic acid; EPS, extracellular polymeric substance; FISH, fluorescent in-situ hybridization; HRT, hydraulic retention time; NGS, next-generation sequencing; OFMSW, organic fraction of municipal solid waste; OLR, organic loading rate; PCR, polymerase chain reaction; PEG, polyethylene glycol; POME: palm oil mill effluent; RNA, ribonucleic acid; RSM, response surface methodology; RT-PCR, reverse transcription polymerase chain reaction; SEM, scanning electron microscopy; UASBR, up-flow anaerobic sludge blanket reactor; VFAs, volatile fatty acids; VS, volatile solids.
